# A phase II clinical study on the efficacy and predictive biomarker of pegylated recombinant arginase on hepatocellular carcinoma

**DOI:** 10.1007/s10637-021-01111-8

**Published:** 2021-04-15

**Authors:** Stephen L. Chan, Paul N.M. Cheng, Angela M. Liu, Landon L. Chan, Leung Li, Cheuk M. Chu, Charing C.N. Chong, Yat M. Lau, Winnie Yeo, Kelvin K.C. Ng, Simon C.H. Yu, Tony S.K. Mok, Anthony W.H. Chan

**Affiliations:** 1 State Key Laboratory of Translational Oncology, Hong Kong, China; 2 Department of Clinical Oncology, Sir YK Pao Centre for Cancer, The Chinese University of Hong Kong, Hong Kong, China; 3 Bio-Cancer Treatment International Ltd., Hong Kong, China; 4 Department of Oncology, Princess Margaret Hospital, Hong Kong, China; 5 Department of Imaging and Interventional Radiology, The Chinese University of Hong Kong, Hong Kong, China; 6 Department of Surgery, The Chinese University of Hong Kong, Hong Kong, China; 7 Department of Anatomical and Cellular Pathology|, The Chinese University of Hong Kong, Hong Kong, China

**Keywords:** Liver cancer, Arginine deprivation, Biomarkers, PEG-BCT-100, Second line

## Abstract

*Background*: Pegylated recombinant human arginase (PEG-BCT-100) is an arginine depleting drug. Preclinical studies showed that HCC is reliant on exogenous arginine for growth due to the under-expression of the arginine regenerating enzymes argininosuccinate synthetase (ASS) and ornithine transcarbamylase (OTC). *Methods*: This is a single arm open-label Phase II trial to assess the potential clinical efficacy of PEG-BCT-100 in chemo naïve sorafenib-failure HCC patients. Pre-treatment tumour biopsy was mandated for ASS and OTC expression by immunohistochemistry (IHC). Weekly intravenous PEG-BCT-100 at 2.7 mg/kg was given. Primary endpoint was time to progression (TTP); secondary endpoints included radiological response as per RECIST1.1, progression free survival (PFS) and overall survival (OS). Treatment outcomes were correlated with tumour immunohistochemical expressions of ASS and OTC. *Results*: In total 27 patients were recruited. The median TTP and PFS were both 6 weeks (95% CI, 5.9–6.0 weeks). The disease control rate (DCR) was 21.7% (5 stable disease). The drug was well tolerated. Post hoc analysis showed that duration of arginine depletion correlated with OS. For patients with available IHC results, 10 patients with ASS-negative tumour had OS of 35 weeks (95% CI: 8.3–78.0 weeks) vs. 15.14 weeks (95% CI: 13.4–15.1 weeks) in 3 with ASS-positive tumour; expression of OTC did not correlate with treatment outcomes. *Conclusions*: PEG-BCT-100 in chemo naïve post-sorafenib HCC is well tolerated with moderate DCR. ASS-negative confers OS advantage over ASS-positive HCC. ASS-negativity is a potential biomarker for OS in HCC and possibly for other ASS-negative arginine auxotrophic cancers. Trial registration number: NCT01092091. Date of registration: March 23, 2010.

**Supplementary Information:**

The online version contains supplementary material available at 10.1007/s10637-021-01111-8.

## Introduction

Arginine is a conditional/semi-essential amino acid involved in the synthesis of a wide range of proteins, a myriad of metabolic pathways as well as being a signing molecule in various cellular pathways [1]. Arginine is also intimately involved in T cell cellular metabolism, T cell receptor (TCR) expression and T cell functionalities [2]. In preclinical studies, arginine deprivation could induce tumour regression in a group of cancers designated as arginine auxotrophic cancers which include melanoma, prostate cancer, acute myeloid leukaemia and hepatocellular carcinoma (HCC) [3, 4]. For selection of cancers for arginine deprivation therapy, it has been postulated that tumours with absent or low expression of arginine regeneration enzymes, argininosuccinate synthase (ASS) and ornithine transcarbamylase (OTC), are reliant on exogenous source of arginine for growth and hence more sensitive to arginine deprivation [3, 5]. In HCC cell lines and patients’ tumour tissues, most of the tumours have no or very low expression of the ASS or OTC on immunohistochemical (IHC) examination [6, 7]. This phenomenon renders HCC a potential cancer type to be benefited from the approach of treatment with arginine deprivation.

PEG-BCT-100 is a pegylated recombinant human arginase 1 protein expressed in *E. coli* which is purified and formulated for human dosing. In clinical trials, PEG-BCT-100 is efficacious in inducing arginine depletion safely and in a dose-dependent manner [8, 9]. In clinical studies, a dose of 2.7 mg/kg/week was found to be the optimal dose of PEG-BCT-100 for sustained arginine depletion for human clinical studies [8]. Regarding evaluation of predictive biomarker for PEG-BCT-100, patients were not mandated to have pre-treatment tumour tissues in previous clinical trials hence evaluations of ASS and OTC were not available to correlate with treatment outcomes [8]. In the present study, we set to evaluate in a phase II setting the treatment efficacy of PEG-BCT-100 in post sorafenib HCC and to correlate treatment outcomes with tumour expression of ASS and OTC (NCT02089763). All patients must have freshly obtained tumour biopsies or archival tumour tissue obtained within 6 months prior to commencement of treatment for analyses of protein expression of ASS and OTC.

## Patients and methods

### Study design and endpoints

This study was an open-label, single-arm Phase II study to evaluate the safety and efficacy of PEG-BCT-100 in chemo naïve patients with prior treatment with sorafenib for HCC. The primary endpoint was time to progression (TTP), which was defined from the time of commencing study treatment (i.e., Cycle 1 Day 1) to the first documentation of objective tumour progression. The secondary endpoints were OS, PFS, radiological response (RR), disease control rate (DCR), type and number of adverse events (AEs), and quality of life (QoL). This clinical trial also explored predictive and prognostic functions of tissue biomarkers with protein expression of ASS and OTC in tumours, as determined by immunohistochemistry. Intended sample size was 30 patients. The study protocol was approved by the Joint Chinese University of Hong Kong-New Territories East Cluster Clinical Research Ethics Committee and was conducted in accordance with the Declaration of Helsinki.

### Eligibility

All patients were recruited at the Prince of Wales Hospital in Hong Kong. Key inclusion criteria included: clinical diagnosis of HCC according to AASLD criteria; age; prior first-line treatment with sorafenib lasting for at least 14 days; adequate haematological and renal function; bilirubin ≤2 x upper limit of normal (ULN) and alanine transaminase (ALT) ≤ 3 x ULN; Eastern Cooperative Oncology Group (ECOG) performance status of 0 or 1. All patients must be fit and willing to undergo a needle biopsy of tumour unless there were archival tumours within 6 months of recruitment to the clinical trial. Key exclusion criteria included: Liver function of Child-Pugh class of B or C; presence of ascites not controlled by medications; prior malignancy and prior treatment with arginine depletion agent.

### Study treatment

Patients were treated with PEG-BCT-100 at 2.7 mg/kg intravenous injection once every week on Day 1, 8 and 15 every 3-weekly cycle. There is no interruption between each cycle. This dosage is equivalent to the dose of 1600 IU/kg weekly administration as used in the previous phase clinical trial [8, 10]. All patients received PEG-BCT-100 till progressive disease, intolerable toxicity, or patients’ consent withdrawal.

### Study assessments

Routine blood tests were measured during screening period, baseline and Day 1 of each 3-weekly cycle. Serum alpha-fetoprotein (AFP) was assessed on day 1 of every cycle. Tumour response was assessed according to the Response Evaluation Criteria In Solid Tumours (RECIST) 1.1 criteria and modified RECIST 1.1 criteria with computed tomography every 6 weeks. Adverse events were evaluated with the Common Terminology Criteria for Adverse Events (CTCAE) 4.0. Quality of life was measured using the European Organization for Research and Treatment of Cancer quality of life questionnaire (EORTC QLQ C-30) and QLQ-HCC18 questionnaires.

Expression of ASS and OTC in the tumour were performed using standard immunohistochemical (IHC) staining protocol with 3, 3′-diaminobenzidine (DAB) as chromogen and polymer amplification. The expression was semi-quantitatively assessed by the Histoscore which was the product of the intensity of staining (graded as: 0, non-staining; 1, weak; 2, median; or 3, strong) and the percentage of positive cells.

### Statistical analyses

Demographic data were summarized by descriptive statistics. Time-to-event data were estimated and plotted with the method of Kaplan-Meier analyses; median estimates and confidence limits will be given. RR and DCR were provided with corresponding exact 95% 2-sided confidence interval with standard methods based on the binomial distribution. Pearson Chi-square test were performed to explore the association between RR and the level of intensity of OTC and ASS. PFS between groups with different level of OTC and/or ASS would be compared by log-rank test, and Cox proportional hazards model were applied for PFS with the biomarkers as predictors. The hazard ratio (HR) and corresponding 95% confidence interval (CI) were estimated. SAS® Software version 9.2 (SAS Institute, Cary, NC, USA).

The sample size and stopping rule was determined using a single-stage design and the number of patients with progressive of disease observed at 2 months. With reference to the previous clinical trial on second-line treatment in HCC, the progression rate at 2-month was around 0.7 for second-line treatment of advanced HCC using placebo (11). Had the progression rate at 2 months been 0.7 or higher, the regimen would have been considered inactive. For a clinical trial with a power higher than 0.8 and a false positive rate of lower than 0.09, the sample size was calculated to be 30.

## Results

### Patient population

Between May 2014 and June 2016, a total of 27 patients were enrolled. Reason for not recruiting the intended sample of 30 patients was due to sponsor’s decision to stop the clinical trial prematurely. Patients’ baseline characteristics were summarized in Table 1. In summary, the median age was 61.1 years, and 24 of them (88.9%) were male. All patients had Child-Pugh class A liver function, with most (96.3%) having Barcelona-Clinic Liver Cancer (BCLC) Stage C disease. The median serum AFP level was 325 ng/ml (Range: 2–420,100 ng/ml; standard deviation: 96742.9).

**Table 1:** Patients’ characteristics

Age (years)	
N	27
Mean	61.09
Standard Deviation	8.651
Minimum, Maximum	46.20, 79.47
Median	61.05
Gender	
Male	24 (88.9%)
Female	3 (11.1%)
ECOG performance status	
Grade 0	13 (48.1%)
Grade 1	13 (48.1%)
Grade 2	1 (3.7%)
Child-Pugh class	
Class A	27 (100.0%)
BCLC stage	
Stage B	1 (3.7%)
Stage C	26 (96.3%)
AFP Level (ng/ml)	
N	27
Mean	27,703
Standard Deviation	96,742.9
Minimum, Maximum	2, 420,100
Median	325

*Abbreviations*: *AFP* alpha-fetoprotein, *BCLC* Barcelona Clinic Liver Cancer, *ECOG* Eastern Cooperative Oncology Group

### Safety and quality of life

The total number of patients included in the safety analysis was 27, and 19 patients (70.4%) had AEs (Supplementary Table 1). The most frequent AE was fatigue (*n* = 16, 59.3%), constipation (*n* = 10, 37.0%) and limb oedema (*n* = 7, 25.9%). For grade 3 or above AEs, commonest events included anaemia (*n* = 2; 7.4%) and hyponatraemia (*n* = 3; 11.1%). All grade 3 or above AEs were considered related to progressive disease by clinical investigators. Treatment interruption was seen in 6 patients (22.2%). Two of them were due to treatment-related AEs (one with fatigue and another due to allergic reaction). No toxicity-related death was observed in the study population. In terms of quality of life, both EORTC QLQ-C30 and EORTC QLQ-HCC18 showed no improvement in symptoms (Supplementary Table 2 and 3). For example, the global health status of EORTC QLQ-30 was 60.9 and 75.4 at baseline and at the end of treatment visit, respectively.

### Efficacy

**Fig. 1:**
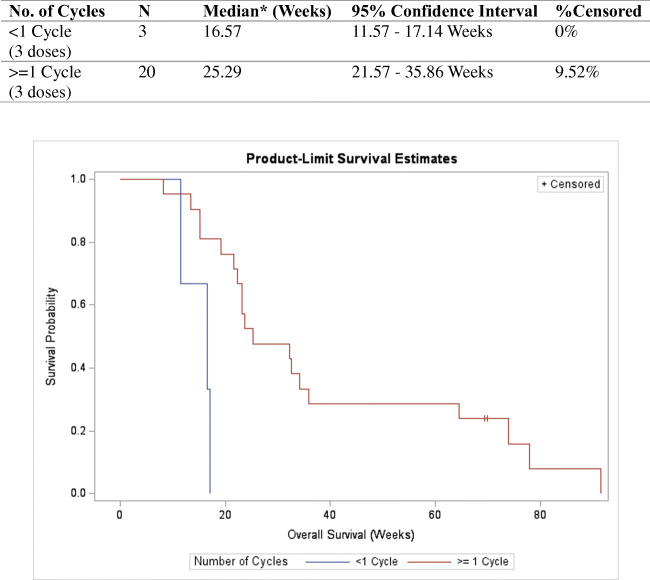
**a** Subgroup analysis of overall survival (OS) by cycle of treatment received

Of the 27 enrolled patients, 23 received at least one dose of the study treatment and had undergone at least one post-dose response assessment. For the primary endpoint, the median TTP was 6.0 weeks (95% CI: 5.9–6.0 weeks) according to RECIST 1.1; the median TTP remained to be 6.0 weeks (95% CI, 6.0–6.1 weeks) when response criteria were changed to mRECIST (Table 2). For secondary efficacy endpoints, 5 patients (21.7%) have stable disease (SD) as the best radiological response amongst the 23 patients. There were no partial or complete responders. The median PFS was 6.0 weeks (95% CI, 5.9–6.0 weeks), and the median OS was 23.7 weeks (95% CI, 19.1–34.1 weeks) (Table 2). Post hoc analysis was performed for 20 patients who received at least 1 cycle (i.e., 3 weekly doses) of study treatment, which achieved adequate duration for complete deprivation of arginine. In this subgroup, the median OS was 16.57 weeks (95% CI: 11.57–17.14 weeks) for patients received <1 cycle of treatment and 25.29 weeks (95% CI: 21.57–35.86 weeks) for patients received ≥1 cycle of treatment (Fig. 1).

**Table 2:** Summary of time-to-progression (TTP), progression-free survival (PFS) and overall survival OS in patients received one dose of treatment (*n* = 23)

	**RECIST 1.1**	**mRECIST**	**OS**
**Time-to-progression (weeks)**			
Subject who progressed	21 (91.3%)	21 (91.3%)	
Number of censoring	2 (8.7%)	2 (8.7%)	
Median time (95% CI)	6.0 (5.86, 6.00)	6.0 (6.00, 6.14)	
Min, Max	3.3, 18.0	3.3, 18.0	
**Progression-free survival (weeks)**			
Subject who progressed/died	22 (95.7%)	23 (100.0%)	
Number of censoring	1 (4.3%)	0 (0.0%)	
Median time (95% CI)	6.0 (5.86, 6.00)	6.0 (6.00, 6.14)	
Min, Max	3.3, 19.1	3.3, 22.3	
**Overall survival (weeks)**			
Subjects who died			21 (91.3%)
Number of censoring			2 (8.7%)
Median time (95% CI)			23.7 (19.14, 34.14)
Min, Max			8.3, 91.6

### Impact of OTC and ASS in tumour tissues

**Fig. 2:**
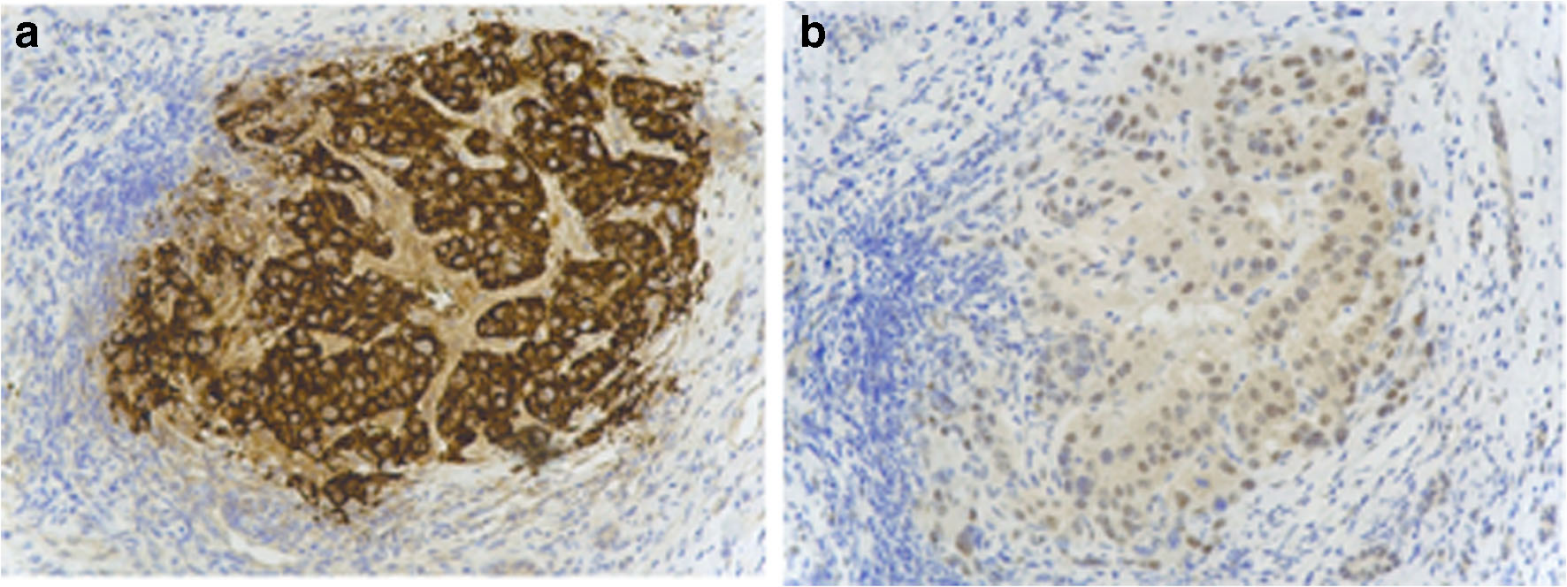
Representative images of immunohistochemical stain for: **a** ornithine transcarbamylase (high expression); **b** arginine succinate synthetase (low expression)

**Fig. 3:**
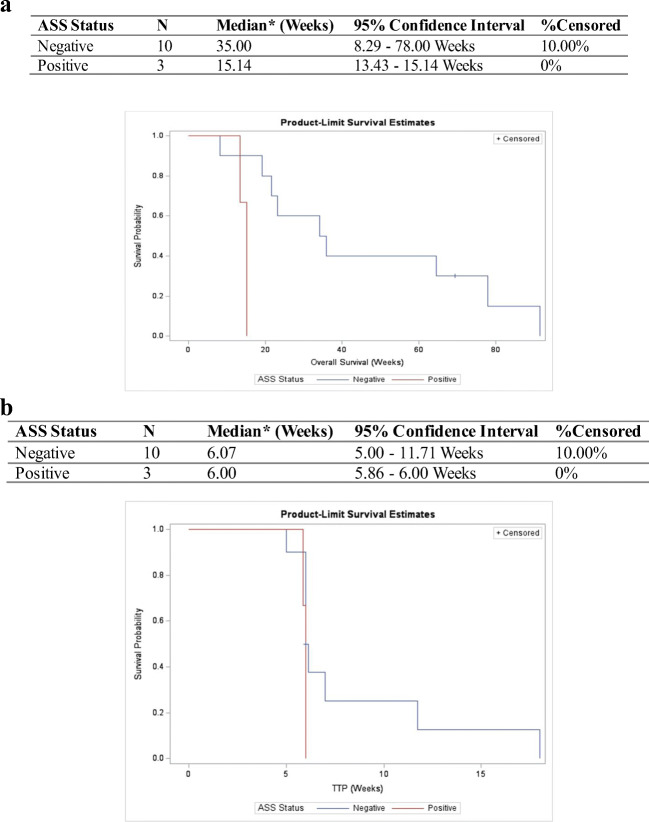
**a** Subgroup analysis of overall survival (OS) by ASS status and **b** time-to-progression (TTP) by ASS status

OTC and ASS expressions in tumour tissues were assessed using IHC. Tissues samples were available for 20 patients, in which 13 patients received at least 1 cycle (i.e., 3 doses) of treatment and undergone post-dose response assessment. For OTC, 3 (23.1%) had No/Low expression (OTC-; histoscore: 0–100); and for ASS, 10 (76.9%) of them had No/Low expression (ASS-NEGATIVE; histoscore: 0–100). Examples of IHC staining of OTC and ASS were illustrated in Fig. 2. The expression levels of OTC and ASS were not associated with radiologically stable disease. For survival, the median OS was 15.1 weeks (95% CI: 12.4–15.1 weeks) and 35.0 weeks (95% CI: 8.3–78.0 weeks) and for 3 patients with ASS+ tumour and 10 patients with ASS-negative tumour (Fig. 3a). Although no significant differences were found in median TTP between ASS+ and ASS-negative groups (6.00 weeks vs. 6.07 weeks), patients with >6 weeks of TTP were all ASS negative (Fig. 3b).

## Discussions

In this Phase II clinical trial, we evaluated the safety, tolerability and efficacy of PEG-BCT-100 in chemo naïve post-sorafenib HCC. Pre-treatment tumour ASS and OTC IHC expression levels were mandated and studied to evaluate the association between these marker expressions and treatment outcomes such as TTP, PFS and OS. The protocol mandated no previous chemo to remove any confounding factors of previous lines of chemotherapy, which may negatively impact on treatment outcomes. As a post-hoc analysis we also studied the OS difference in those patients who had arginine depletion with PEG-BCT-100 over one cycle.

As with the previous published data on PEG-BCT-100, results of current study confirm the safety and tolerability of PEG-BCT-100 [10]. Although the clinical trial failed its primary endpoint in extending TTP to beyond 2 months, there are nevertheless two clinically relevant findings that warrant discussion. First, ASS-negative tumour confers OS advantage over ASS-positive tumour: patients with ASS-negative tumour were found to have doubled the OS compared to the ASS+ ones (35 versus 15 weeks). On the contrary, the OTC status, whether positive or negative, were found not to associate with tumour response or OS. These data suggest that ASS but not the OTC expression is a more clinically relevant biomarker for PEG-BCT-100, and ASS negativity should be evaluated as a patient enrichment strategy in future clinical studies on PEG-BCT-100. Second, the duration of arginine depletion is found to be crucial for clinical benefits of arginine deprivation. In the current study, an OS advantage was demonstrated in patients with PEG-BCT-100 treatment >3 weeks (25.29 weeks vs. 16.57 weeks). This OS advantage in prolonged arginine depletion with PEG-BCT-100 is consistent with the two studies of ADI-peg, which is a pegylated arginine deiminase. In the ADI-peg global HCC phase III study [11], the authors reported OS advantage for those patients who had arginine depletion over 6 weeks. In another Asian ADI-peg HCC study [12], it was similarly found that OS benefits were only observed in patients who had arginine depletion >3 weeks as compared to those with <3 weeks. Above findings all indicate that 3 to 6 weeks is an optimal duration of clinically meaningful arginine deprivation, which is important for clinical trial design on arginine deprivation.

The survival advantage in ASS-negative HCC could have implication in the treatment of other ASS-negative tumours since a number of common tumours are arginine auxotrophic by virtual of the negative ASS status, including melanoma, prostate cancer, neuroblastoma, acute myeloid leukaemia, glioma and some sarcoma [7]. In the US study of PET-BCT-100, it was shown that durable complete remission was observed in a patient with metastatic melanoma which was subsequently found to have negative ASS expression on immunohistochemistry [9]. Similar observation was made with another arginine depletion treatment: amongst 9 patients with ASS-negative thoracic cancers were given chemotherapy and ADI-peg, 7 of them had partial responses [13]. To further explore above concept in other cancer types, a number of clinical trials are ongoing, including the Phase II clinical trials on PEG-BCT-100 either singly in paediatrics cancers such as neuroblastoma and glioma (EUDRACT no:2017–002762-44) and another clinical trial on PEG-BCT-100 in combination with low dose cytarabine and in acute myeloid leukaemia (EUDRACT no:2011–000749).

About the potential clinical application, it is understood that tyrosine kinase inhibitors and check-point inhibitor immunotherapy are the current standard treatment for HCC. However, personalized treatment with biomarker for selection remains elusive for HCC. Our study provide preliminary evidence on applying ASS-negativity in tumour tissues as a biomarker for selection of patients for arginine deprivation therapy. There remains room for developing this strategy as monotherapy in later lines of systemic treatment for HCC. Further, the combination of arginine deprivation with other anti-cancer treatment could improve the efficacy in ASS-negative HCC [14]. Hence it is clinically important to evaluate combinational strategy on PEG-BCT-100 with other existing treatments in HCC. Finally, the existent and emerging immune-oncology treatments are associated with high cost and the potential occurrence of hyperprogression in HCC [15, 16]. For HCC patients who fail or could not afford targeted agents/immunotherapy, arginine depletion therapy with PEG-BCT-100 could still be an attractive and affordable treatment option.

In conclusion, the current phase II clinical trial shows that PEG-BCT-100 monotherapy is well tolerated in second-line post-sorafenib HCC, with moderate anti-tumour activity. In the first time, it is shown in human that ASS-negativity in tumour confers survival advantage over ASS positive patients, which support the potential role of ASS-negative as a predictive biomarker for patient selection. Further studies are now underway to further confirm ASS-negative as a predictive OS biomarker for treatment of arginine auxotrophic cancers with PEG-BCT-100.

## Supplementary information

ESM 1 (PDF 125 kb)
