# Supplementary material for: A phase II clinical study on the efficacy and predictive biomarker of pegylated recombinant arginase on hepatocellular carcinoma
Source: Invest New Drugs. 2021 Apr 15;39(5):1375–82. doi: 10.1007/s10637-021-01111-8 (PMC8426309; doi:10.1007/s10637-021-01111-8)
Supplement: Supplementary file 1 — (PDF 125 kb) [file 10637_2021_1111_MOESM1_ESM.pdf]

Supplementary Table 1. Incidence and severity of adverse events

	All Grade	Grade 1	Grade 2	Grade 3	Grade 4
**Patients with AE(s)**	**27 (100.0)**	**26 (96.3)**	**19 (70.4)**	**8 (29.6)**	**1 (3.7)**
**Blood and lymphatic system disorders**	**2 (7.4)**	**0**	**0**	**2 (7.4)**	**0**
Anaemia	2 (7.4)	0	0	2 (7.4)	0
**Cardiac disorders**	**2 (7.4)**	**1 (3.7)**	**1 (3.7)**	**0**	**0**
Palpitations	1 (3.7)	1 (3.7)	0	0	0
Sinus tachycardia	1 (3.7)	0	1 (3.7)	0	0
**Endocrine disorders**	**1 (3.7)**	**0**	**1 (3.7)**	**0**	**0**
Hypothyroidism	1 (3.7)	0	1 (3.7)	0	0
**Gastrointestinal disorders**	**16 (59.3)**	**12 (44.4)**	**8 (29.6)**	**2 (7.4)**	**0**
Abdominal distension	1 (3.7)	0	1 (3.7)	0	0
Abdominal pain	3 (11.1)	0	3 (11.1)	0	0
Ascites	3 (11.1)	1 (3.7)	2 (7.4)	0	0
Constipation	10 (37.0)	7 (25.9)	3 (11.1)	0	0
Diarrhoea	3 (11.1)	3 (11.1)	0	0	0
Duodenal haemorrhage	1 (3.7)	0	0	1 (3.7)	0
Dyspepsia	5 (18.5)	3 (11.1)	2 (7.4)	0	0
Oesophageal ulcer	1 (3.7)	0	1 (3.7)	0	0
Oesophageal varices haemorrhage	1 (3.7)	0	0	1 (3.7)	0
Gastrointestinal disorders – Other, abdominal colic	1 (3.7)	1 (3.7)	0	0	0
Mucositis oral	1 (3.7)	1 (3.7)	0	0	0
Nausea	6 (22.2)	3 (11.1)	3 (11.1)	0	0
Vomiting	2 (7.4)	2 (7.4)	0	0	0
**General disorders and administration site conditions**	**22 (81.5)**	**20 (74.1)**	**8 (29.6)**	**0**	**0**
Oedema limbs	7 (25.9)	7 (25.9)	0	0	0
Fatigue	16 (59.3)	13 (48.1)	5 (18.5)	0	0
Fever	6 (22.2)	5 (18.5)	2 (7.4)	0	0
Malaise	1 (3.7)	1 (3.7)	0	0	0
Non-cardiac chest pain	1 (3.7)	0	1 (3.7)	0	0
**Hepatobiliary disorders**	**4 (14.8)**	**1 (3.7)**	**1 (3.7)**	**2 (7.4)**	**0**
Hepatobiliary disorders – Other, obstructive jaundice	2 (7.4)	0	0	2 (7.4)	0
Hepatobiliary disorders – Other, tumour rupture	1 (3.7)	0	1 (3.7)	0	0
Hepatobiliary disorders, Other – hepatomegaly	1 (3.7)	1 (3.7)	0	0	0
**Immune system disorders**	**1 (3.7)**	**0**	**1 (3.7)**	**0**	**0**
Allergic reaction	1 (3.7)	0	1 (3.7)	0	0
**Infections and infestations**	**3 (11.1)**	**1 (3.7)**	**2 (7.4)**	**0**	**0**

Infections and infestations – Other, pulmonary tuberculosis	1 (3.7)	0	1 (3.7)	0	0
Upper respiratory infection	2 (7.4)	1 (3.7)	1 (3.7)	0	0
**Injury, poisoning and procedural complications**	**1 (3.7)**	**1 (3.7)**	**0**	**0**	**0**
Bruising	1 (3.7)	1 (3.7)	0	0	0
**Investigations**	**8 (29.6)**	**4 (14.8)**	**3 (11.1)**	**1 (3.7)**	**1 (3.7)**
Alanine aminotransferase increased	1 (3.7)	1 (3.7)	0	0	0
Alkaline phosphatase increased	1 (3.7)	0	0	1 (3.7)	0
Blood bilirubin increased	2 (7.4)	0	1 (3.7)	1 (3.7)	1 (3.7)
Creatinine increased	1 (3.7)	0	0	1 (3.7)	0
Platelet count decreased	1 (3.7)	1 (3.7)	0	0	0
Weight gain	2 (7.4)	0	2 (7.4)	0	0
Weight loss	2 (7.4)	2 (7.4)	0	0	0
**Metabolism and nutrition disorders**	**8 (29.6)**	**4 (14.8)**	**6 (22.2)**	**3 (11.1)**	**0**
Anorexia	2 (7.4)	1 (3.7)	1 (3.7)	0	0
Hypercalcemia	1 (3.7)	0	1 (3.7)	0	0
Hyperglycaemia	1 (3.7)	1 (3.7)	0	0	0
Hyperkalaemia	1 (3.7)	0	1 (3.7)	0	0
Hypoalbuminemia	1 (3.7)	0	1 (3.7)	0	0
Hypokalaemia	4 (14.8)	1 (3.7)	3 (11.1)	0	0
Hyponatremia	4 (14.8)	1 (3.7)	0	3 (11.1)	0
Hypophosphatemia	1 (3.7)	0	0	1 (3.7)	0
**Musculoskeletal and connective tissue disorders**	**3 (11.1)**	**0**	**3 (11.1)**	**0**	**0**
Back pain	1 (3.7)	0	1 (3.7)	0	0
Musculoskeletal and connective tissue disorder – Other, left shoulder pain	1 (3.7)	0	1 (3.7)	0	0
Pain in extremity	1 (3.7)	0	1 (3.7)	0	0
**Neoplasms benign, malignant and unspecified (including cysts and polyps)**	**1 (3.7)**	**1 (3.7)**	**0**	**0**	**0**
Neoplasms benign, malignant and unspecified (including cysts and polyps) – Other, Benign prostatic hyperplasia	1 (3.7)	1 (3.7)	0	0	0
**Nervous system disorders**	**3 (11.1)**	**0**	**1 (3.7)**	**2 (7.4)**	**0**
Nervous system disorders – Other, cord compression	2 (7.4)	0	0	2 (7.4)	0
Nervous system disorders – Other, sciatica	1 (3.7)	0	1 (3.7)	0	0
**Psychiatric disorders**	**5 (18.5)**	**3 (11.1)**	**2 (7.4)**	**0**	**0**
Insomnia	5 (18.5)	3 (11.1)	2 (7.4)	0	0

**Renal and urinary disorders**	**4 (14.8)**	**3 (11.1)**	**2 (7.4)**	**0**	**0**
Renal and urinary disorders – Other, dysuria	3 (11.1)	3 (11.1)	0	0	0
Urinary retention	2 (7.4)	0	2 (7.4)	0	0
**Respiratory, thoracic and mediastinal disorders**	**12 (44.4)**	**12 (44.4)**	**4 (14.8)**	**1 (3.7)**	**0**
Cough	5 (18.5)	5 (18.5)	0	0	0
Dyspnoea	3 (11.1)	3 (11.1)	0	0	0
Epistaxis	1 (3.7)	1 (3.7)	0	0	0
Hiccups	1 (3.7)	0	1 (3.7)	0	0
Pleural effusion	2 (7.4)	2 (7.4)	1 (3.7)	0	0
Pneumothorax	1 (3.7)	0	1 (3.7)	0	0
Productive cough	5 (18.5)	4 (14.8)	1 (3.7)	0	0
Respiratory, thoracic and mediastinal disorders – Other, haemoptysis	1 (3.7)	0	0	1 (3.7)	0
Respiratory, thoracic and mediastinal disorders – Other, runny nose	1 (3.7)	1 (3.7)	0	0	0
Sore throat	3 (11.1)	2 (7.4)	1 (3.7)	0	0
**Skin and subcutaneous tissue disorders**	**5 (18.5)**	**5 (18.5)**	**0**	**0**	**0**
Alopecia	2 (7.4)	2 (7.4)	0	0	0
Dry skin	1 (3.7)	1 (3.7)	0	0	0
Rash maculo-papular	3 (11.1)	3 (11.1)	0	0	0
Skin and subcutaneous tissue disorders – Other, bed sore	1 (3.7)	1 (3.7)	0	0	0
**Vascular disorders**	**1 (3.7)**	**1 (3.7)**	**0**	**1 (3.7)**	**0**
Hematoma	1 (3.7)	1 (3.7)	0	0	0
Thromboembolic event	1 (3.7)	0	0	1 (3.7)	0

(Abbreviations: AE, adverse events)

Supplementary Table 2. Quality of life (EORTC QLQ-C30) at the baseline and end of treatment visit

**Global health status**		
Baseline		
	N	23
	Mean (SD)	60.87 (20.480)
	Median (Q1, Q3)	58.33 (50.00, 66.67)
	Minimum, Maximum	33.33, 100.00
EoT Visit		
	N	22
	Mean (SD)	54.55 (25.938)
	Median (Q1, Q3)	58.33 (33.33, 66.67)
	Minimum, Maximum	0.00, 100.00
**Physical functioning**		
Baseline		
	N	23
	Mean (SD)	75.36 (15.100)
	Median (Q1, Q3)	80.00 (60.00, 86.67)
	Minimum, Maximum	46.67, 100.00
EoT Visit		
	N	22
	Mean (SD)	60.23 (29.922)
	Median (Q1, Q3)	70.00 (33.33, 86.67)
	Minimum, Maximum	0.00, 100.00
**Role functioning**		
Baseline		
	N	23
	Mean (SD)	71.01 (28.523)
	Median (Q1, Q3)	66.67 (50.00, 100.00)
	Minimum, Maximum	0.00, 100.00
EoT Visit		
	N	22
	Mean (SD)	65.15 (38.459)
	Median (Q1, Q3)	66.67 (50.00, 91.67)
	Minimum, Maximum	0.00, 100.00
**Emotional functioning**		
Baseline		
	N	23
	Mean (SD)	77.17 (20.752)
	Median (Q1, Q3)	66.67 (66.67, 100.00)
	Minimum, Maximum	41.67, 100.00
EoT Visit		
	N	22
	Mean (SD)	72.73 (19.616)
	Median (Q1, Q3)	66.67 (66.67, 91.67)
	Minimum, Maximum	41.67, 100.00
**Cognitive functioning**		
Baseline		
	N	23
	Mean (SD)	82.61 (14.632)

Median (Q1, Q3)	83.33 (66.67, 100.00)
Minimum, Maximum	66.67, 100.00
EoT Visit	
N	22
Mean (SD)	73.48 (20.993)
Median (Q1, Q3)	66.67 (66.67, 100.00)
Minimum, Maximum	33.33, 100.00
**Social Functioning**	
Baseline	
N	23
Mean (SD)	73.91 (24.009)
Median (Q1, Q3)	66.67 (66.67, 100.00)
Minimum, Maximum	33.33, 100.00
EoT Visit	
N	22
Mean (SD)	68.18 (31.248)
Median (Q1, Q3)	66.67 (66.67, 100.00)
Minimum, Maximum	0.00, 100.00
**Fatigue**	
Baseline	
N	23
Mean (SD)	31.88 (22.549)
Median (Q1, Q3)	33.33 (22.22, 33.33)
Minimum, Maximum	0.00, 88.89
EoT Visit	
N	22
Mean (SD)	46.46 (30.213)
Median (Q1, Q3)	38.89 (22.22, 66.67)
Minimum, Maximum	0.00, 100.00
**Nausea and vomiting**	
Baseline	
N	23
Mean (SD)	9.42 (15.752)
Median (Q1, Q3)	0.00 (0.00, 16.67)
Minimum, Maximum	0.00, 50.00
EoT Visit	
N	22
Mean (SD)	17.42 (24.385)
Median (Q1, Q3)	0.00 (0.00, 33.33)
Minimum, Maximum	0.00, 83.33
**Pain**	
Baseline	
N	23
Mean (SD)	29.71 (24.596)
Median (Q1, Q3)	33.33 (0.00, 50.00)
Minimum, Maximum	0.00, 66.67
EoT Visit	
N	22
Mean (SD)	39.39 (29.343)
Median (Q1, Q3)	33.33 (16.67, 66.67)

Minimum, Maximum	0.00, 100.00
**Dyspnoea**	
Baseline	
N	23
Mean (SD)	24.64 (22.957)
Median (Q1, Q3)	33.33 (0.00, 33.33)
Minimum, Maximum	0.00, 66.67
EoT Visit	
N	22
Mean (SD)	36.36 (36.960)
Median (Q1, Q3)	33.33 (0.00, 66.67)
Minimum, Maximum	0.00, 100.00
**Insomnia**	
Baseline	
N	23
Mean (SD)	36.23 (30.005)
Median (Q1, Q3)	33.33 (0.00, 66.67)
Minimum, Maximum	0.00, 100.00
EoT Visit	
N	22
Mean (SD)	37.88 (33.007)
Median (Q1, Q3)	33.33 (0.00, 66.67)
Minimum, Maximum	0.00, 100.00
**Appetite loss**	
Baseline	
N	23
Mean (SD)	26.09 (31.713)
Median (Q1, Q3)	0.00 (0.00, 66.67)
Minimum, Maximum	0.00, 100.00
EoT Visit	
N	22
Mean (SD)	43.94 (40.351)
Median (Q1, Q3)	33.33 (0.00, 66.67)
Minimum, Maximum	0.00, 100.00
**Constipation**	
Baseline	
N	23
Mean (SD)	11.59 (16.233)
Median (Q1, Q3)	0.00 (0.00, 33.33)
Minimum, Maximum	0.00, 33.33
EoT Visit	
N	22
Mean (SD)	18.18 (28.595)
Median (Q1, Q3)	0.00 (0.00, 33.33)
Minimum, Maximum	0.00, 100.00
**Diarrhoea**	
Baseline	
N	23
Mean (SD)	13.04 (21.879)
Median (Q1, Q3)	0.00 (0.00, 33.33)

Minimum, Maximum	0.00, 66.67
EoT Visit	
N	22
Mean (SD)	12.12 (24.224)
Median (Q1, Q3)	0.00 (0.00, 0.00)
Minimum, Maximum	0.00, 66.67
**Financial difficulties**	
Baseline	
N	23
Mean (SD)	47.83 (31.505)
Median (Q1, Q3)	33.33 (33.33, 66.67)
Minimum, Maximum	0.00, 100.00
EoT Visit	
N	22
Mean (SD)	43.94 (29.790)
Median (Q1, Q3)	33.33 (33.33, 66.67)
Minimum, Maximum	0.00, 100.00

(Abbreviations: EoT, end of treatment; SD, standard deviation)

Supplementary Table 3. Quality of life (EORTC QLQ-HCC18) at the baseline and end of treatment visit

**Fatigue symptom scale**	
Baseline	
N	20
Mean (SD)	35.83 (25.265)
Median (Q1, Q3)	33.33 (11.11, 52.78)
Minimum, Maximum	0.00, 88.89
EoT Visit	
N	22
Mean (SD)	36.87 (26.201)
Median (Q1, Q3)	38.89 (11.11, 44.44)
Minimum, Maximum	0.00, 88.89
**Body image scale**	
Baseline	
N	20
Mean (SD)	24.17 (23.863)
Median (Q1, Q3)	33.33 (0.00, 33.33)
Minimum, Maximum	0.00, 83.33
EoT Visit	
N	22
Mean (SD)	28.79 (20.041)
Median (Q1, Q3)	33.33 (16.67, 33.33)
Minimum, Maximum	0.00, 83.33
**Jaundice symptom scale**	
Baseline	
N	20
Mean (SD)	20.00 (21.357)
Median (Q1, Q3)	16.67 (0.00, 33.33)
Minimum, Maximum	0.00, 66.67
EoT Visit	
N	22
Mean (SD)	18.18 (17.750)
Median (Q1, Q3)	16.67 (0.00, 33.33)
Minimum, Maximum	0.00, 66.67
**Nutrition scale**	
Baseline	
N	20
Mean (SD)	26.67 (24.086)
Median (Q1, Q3)	30.00 (0.00, 43.33)
Minimum, Maximum	0.00, 73.33
EoT Visit	
N	22
Mean (SD)	33.03 (27.639)
Median (Q1, Q3)	26.67 (13.33, 53.33)
Minimum, Maximum	0.00, 86.67
**Pain scale**	
Baseline	
N	20
Mean (SD)	24.17 (27.823)

Median (Q1, Q3)	16.67 (0.00, 33.33)
Minimum, Maximum	0.00, 100.00
EoT Visit	
N	22
Mean (SD)	25.76 (24.520)
Median (Q1, Q3)	25.00 (0.00, 33.33)
Minimum, Maximum	0.00, 83.33
**Fever scale**	
Baseline	
N	20
Mean (SD)	5.00 (10.949)
Median (Q1, Q3)	0.00 (0.00, 0.00)
Minimum, Maximum	0.00, 33.33
EoT Visit	
N	22
Mean (SD)	13.64 (20.339)
Median (Q1, Q3)	0.00 (0.00, 16.67)
Minimum, Maximum	0.00, 66.67
**Abdominal swelling**	
Baseline	
N	20
Mean (SD)	16.67 (27.572)
Median (Q1, Q3)	0.00 (0.00, 33.33)
Minimum, Maximum	0.00, 100.00
EoT Visit	
N	22
Mean (SD)	24.24 (25.577)
Median (Q1, Q3)	33.33 (0.00, 33.33)
Minimum, Maximum	0.00, 100.00
**Sex life**	
Baseline	
N	17
Mean (SD)	35.29 (36.268)
Median (Q1, Q3)	33.33 (0.00, 66.67)
Minimum, Maximum	0.00, 100.00
EoT Visit	
N	19
Mean (SD)	43.86 (41.652)
Median (Q1, Q3)	33.33 (0.00, 100.00)
Minimum, Maximum	0.00, 100,00

(Abbreviations: EoT, end of treatment; SD, standard deviation)
